# An Unusual Presentation of Baker's Cyst Rupture in an Inflammatory Bowel Disease Patient

**DOI:** 10.1155/2020/3149058

**Published:** 2020-01-07

**Authors:** Gianna Stoleru, Lauren George, Raymond K. Cross, Uni Wong

**Affiliations:** University of Maryland School of Medicine, Department of Medicine, Division of Gastroenterology and Hepatology, Baltimore, MD, USA

## Abstract

When evaluating a patient with acute onset unilateral leg pain and concurrent inflammatory bowel disease (IBD), keeping a broad differential diagnosis will allow for prompt diagnosis and management. The patient described in this case report is a 32-year-old male with inflammatory ileocolonic Crohn's disease (CD) status after ileocecectomy with perianal involvement and known Type 1 arthropathy. He presented with a three-day history of unilateral leg swelling and tenderness. Initial evaluation focused on possible thrombosis given the development of erythema and systemic symptoms. Final diagnosis was ruptured Baker's (popliteal) cyst. This pathology is not well described in existing literature, but should be considered in IBD patients given their chronic inflammatory state and common associated intra-articular pathology.

## 1. Introduction

This case describes a ruptured Baker's cyst in a patient with ileocolonic Crohn's disease (CD) in an effort to aid the clinician in prompt diagnosis of acute unilateral leg pain in patients with inflammatory bowel disease (IBD). This pathology occurs when fluid accumulates between the bursa of the gastrocnemius muscle and semimembranosus tendon [1]. This is not well described in existing literature of patients with IBD. It has anecdotally been associated with other inflammatory states, such as rheumatoid arthritis [2] and infection [3].

## 2. Case Report

The patient is a 32-year-old male with history of inflammatory ileocolonic Crohn's disease (CD) status after ileocecectomy with perianal involvement, Type 1 arthropathy with synovitis of the bilateral knees, essential hypertension, recurrent cellulitis, and obesity, who presented to our institution for ustekinumab therapy. At that time, he reported a three-day history of right knee pain and swelling. He reported that this initial presentation was similar to past arthropathy flares. The patient had a known history of spondyloarthropathy, best explained as an extraintestinal manifestation of his active CD. The patient noted development of intermittent fevers and chills as well. During his ustekinumab infusion, the patient had acute worsening of his knee pain with sudden extension into the right calf, ankle joint, and foot. He also reported tightness and tenderness to palpation of the posterior right calf. This specifically, as well as the development of systemic symptoms, differed from his prior Type 1 arthropathy symptoms in the affected extremity.

**Figure 1:**
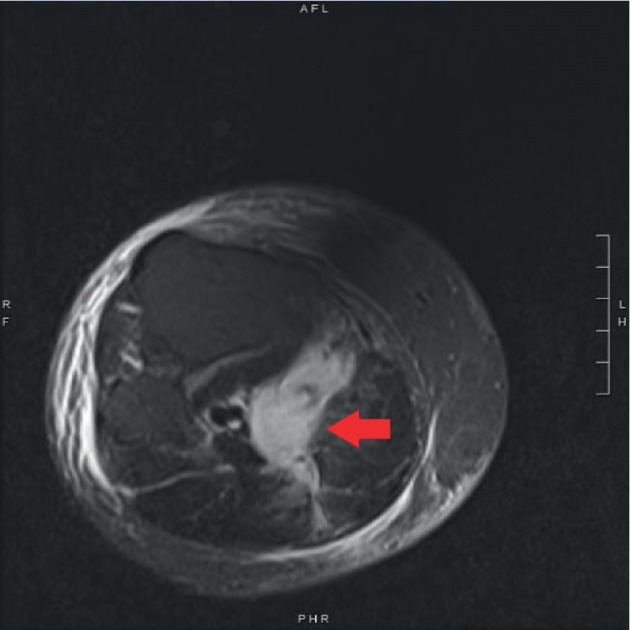
MRI imaging demonstrating complex fluid in the semimembranosus-gastrocnemius bursa (red arrow) with associated edema. Surrounding fluid extravasation is representative of ruptured Baker's cyst. Associated subcutaneous, muscular, and fascial edema is visualized.

Upon presentation to the Emergency Department, his pain had progressed in severity with associated nausea. The affected extremity had also developed erythema and had become exquisitely tender to palpation. He denied cough, shortness of breath, abdominal pain, headache, lightheadedness, or dizziness. The patient was tachycardic to 136 beats per minute and febrile to 38° Celsius. He was treated with analgesics, with notable labs including WBC of 14.4 × 10^3^/*μ*L, K+ of 3.3 mEq/L, Cr of 0.63 mg/dL, and CK 21 units/L. Blood draw and knee joint aspiration for cultures were both negative. Empiric antibiotic therapy was started, and Orthopedics was consulted for concern of compartment syndrome. The patient was not found to have developed compartment syndrome and maintained distal pulses identifiable on Doppler. The affected extremity remained warm and appeared well perfused. Radiographs of the lower extremity were unremarkable. Ultrasound venous duplex was completed to evaluate for deep vein thrombosis (DVT) given the development of erythema and systemic symptoms, and was negative. On ultrasound, soft tissue swelling of the posterior calf with incidental Baker's cyst in the medial aspect of the calf, measuring 6.8 × 4.0 cm, was noted in addition to small joint effusion. Further evaluation with lower extremity MRI showed evidence of complex fluid in the right semimembranosus-gastrocnemius bursa with edema suggestive of extravasation of fluid from rupture of Baker's cyst (Figure 1). There was no evidence of osteomyelitis. A CT pelvis was performed to rule out other causes of lower extremity edema.

The patient was maintained on acetaminophen and oxycodone for pain control. Orthopedics recommended weight bearing and range of motion as tolerated, and antibiotics were discontinued. Rheumatology was consulted and recommended outpatient intra-articular cortisone injection for persistent swelling and pain. The patient was continued on his home doses of prednisone and oral methotrexate. Upon 2-month follow-up in the inflammatory bowel disease (IBD) clinic, the patient had not pursued outpatient cortisone injection. He reported complete resolution of the acute symptoms related to rupture of Baker's cyst, which he treated conservatively with acetaminophen. At the time of follow-up, he reported persistent pain related to his known Type 1 arthropathy in the bilateral knees, which he described as predominantly stiffness without swelling.

## 3. Discussion

Inflammatory bowel disease (IBD) is a chronic inflammatory condition of the gastrointestinal (GI) tract that includes both Crohn's disease (CD) and ulcerative colitis (UC). While the exact pathogenesis of IBD is uncertain, it is commonly understood that disease results from poorly regulated and inconsistent mucosal reaction to environmental insult in genetically predisposed patients [4].

While most obvious symptomology of IBD is GI in nature, patients commonly present with extraintestinal manifestations (EIMs). EIMs present as primary complaints in patients with no known history of IBD, or over time in a patient with an established IBD diagnosis. The most commonly affected sites outside of the GI tract include the joints, eyes, skin, and hepatobiliary system [5]. One study by Isene et al. describes that patients with CD are two times as likely to experience EIMs compared to patients with UC [5]. Additionally, CD patients most commonly experience arthritis [5].

IBD patients commonly present with joint and musculoskeletal complaints, with a subsequent broad differential of etiologies. An established common cause of acute unilateral leg pain in patients with IBD is deep vein thrombosis (DVT). This association is well supported by existing literature, which describes the role of inflammation in tending to shift the hemostatic balance towards a thrombotic state via activation of coagulation factors, which worsens the overall condition by sustaining inflammation [6–8]. Many clinicians are aware of this association; however, alternate diagnoses may act as mimics, including Baker's cyst.

A Baker's cyst forms when there is excess synovial fluid or synovitis present that is compressed by the body weight and surrounding structures. Separation from the intra-articular compartment eventually occurs, and an independent sac or cyst forms. Intra-articular pathology, such as meniscal tears and osteoarthritis, are commonly associated with formation of Baker's cysts. However, ruptured Baker's cysts have also been associated with inflammatory conditions including rheumatoid arthritis and gout [1, 2, 9, 10]. This patient's known Type 1 arthropathy, with synovitis, increased his risk of developing Baker's cyst given the natural mechanism of development. Joint inflammation from this patient's IBD-associated spondyloarthropathy, including the affected joint, likely caused localized inflammation and soft tissue swelling that may have contributed to overall compartment pressure and eventual rupture.

A palpable, symptomatic enlargement of the gastrocnemius-semimembranosus bursa defines a Baker's cyst on physical exam [11]. The presentation of a ruptured Baker's cyst is typically acute with associated moderate to severe unilateral leg pain, calf fullness, and worsening pain on extension of the joint or other physical activity [1]. Erythema of the affected extremity and associated systemic symptoms including fever, chills, and tachycardia, all of which were present in the case described, are not typical in presentation of a ruptured Baker's cyst.

When assessing unilateral leg pain in patients with IBD, inflammatory arthritis can also be considered [12]. Peripheral arthropathies in IBD are relatively common, occurring in approximately 5–20% of patients with IBD and 10–20% in CD specifically [12]. Imaging studies of IBD-associated arthritis are unique in that they do not show gross deformity, in contrast to other forms of arthritis such as osteoarthritis or rheumatoid arthritis.

While multimodal imaging may help in distinguishing ruptured Baker's cyst from other lower extremity pathology in the setting of IBD flare, it is not necessary for diagnosis. Relatively fast and inexpensive imaging, such as ultrasound, should demonstrate a sonolucent area behind the muscles of the mid-calf [11, 13]. This useful sign found on ultrasound can help direct immediate therapy and is sufficient in making the diagnosis in the appropriate clinical context.

This case demonstrates the importance of distinguishing between the many musculoskeletal EIMs of IBD that can present as acute unilateral leg pain. Most interestingly, this case highlights an unusual presentation of a ruptured Baker's cyst, a pathology not well described in IBD patients, and how basic imaging such as ultrasound can provide efficient differentiation from DVT in the setting of atypical systemic symptoms.
